# Thiran Filters for Wideband DSP-Based Multi-Beam True Time Delay RF Sensing Applications

**DOI:** 10.3390/s24020576

**Published:** 2024-01-17

**Authors:** Sirani M. Perera, Gayani Rathnasekara, Arjuna Madanayake

**Affiliations:** 1 Department of Mathematics, Embry-Riddle Aeronautical University, Daytona Beach, FL 32114, USA; 2 Department of Electrical and Computer Engineering, Florida International University, Miami, FL 33199, USA; grath003@fiu.edu (G.R.); amadanay@fiu.edu (A.M.)

**Keywords:** complexity and performance of algorithm, structured matrices, signal processing and analysis, TTD wideband multi-beam beamforming, Thiran fractional delays, array processing, wireless communication

## Abstract

The ability to sense propagating electromagnetic plane waves based on their directions of arrival (DOAs) is fundamental to a range of radio frequency (RF) sensing, communications, and imaging applications. This paper introduces an algorithm for the wideband true time delay digital delay Vandermonde matrix (DVM), utilizing Thiran fractional delays that are useful for realizing RF sensors having multiple look DOA support. The digital DVM algorithm leverages sparse matrix factorization to yield multiple simultaneous RF beams for low-complexity sensing applications. Consequently, the proposed algorithm offers a reduction in circuit complexity for multi-beam digital wideband beamforming systems employing Thiran fractional delays. Unlike finite impulse response filter-based approaches which are wideband but of a high filter order, the Thiran filters trade usable bandwidth in favor of low-complexity circuits. The phase and group delay responses of Thiran filters with delays of a fractional sampling period will be demonstrated. Thiran filters show favorable results for sample delay blocks with a temporal oversampling factor of three. Thiran fractional delays of orders three and four are mapped to Xilinx FPGA RF-SoC technologies for evaluation. The preliminary results of the APF-based Thiran fractional delays on FPGA can potentially be used to realize DVM factorizations using application-specific integrated circuit (ASIC) technology.

## 1. Introduction

The directional enhancement of propagating RF plane waves is a key requirement for electromagnetic sensing using antenna array processors. Linear arrays of uniformly spaced wideband antenna elements receive far-field propagating electromagnetic plane waves, which must be directionally enhanced along the desired look direction using a beamformer. A TTD beamformer enhances a wideband plane-wave signal along a particular DOA. To wit, as the instantaneous bandwidth of modern antenna apertures increases, it leads to wideband systems. The underlying beamforming schemes for such an aperture must be realized with support for squint-free operation across the bands of interest [1]. The TTD beamformer is the optimal beamformer when the signals of interest are contaminated by wide sense stationary AWGN [2,3,4,5]; the case of the sensing of RF plane waves along multiple directions is a crucially important application for antenna array-based RF sensing and direction-of-arrival estimation [6] with use cases in RF imaging, RF communications, and RF location.

For an aperture with *N* elements, an *N*-th order TTD beamformer can be realized using analog, mixed-signal, or direct-digital methods for realizing such a TTD aperture beam. A TTD beamformer can be steered by the appropriate selection of the progressive time delay from one element to the next in a uniform aperture configuration. In cases where a large number of wideband beams are required, a dedicated TTD beam must be created through its own TTD beamsteering network [5]. Typically, for *N* elements, *N*-independent beams can be realized by considering a short duration time delay $\tau_{0}=T/N$, where *T* is the time taken for the radio signal to propagate between two neighboring elements in the aperture. The parallel realization of *N* beams using a network of TTDs with durations set to integer multiples of $\tau_{0}$ leads to an *N*-beam TTD multi-beam beamformer with an underlying mathematical framework given by the DVM [7,8,9,10,11]. The DVM is similar in concept to a spatial DFT; however, unlike the case of the DFT which uses a fixed complex coefficient in the matrix–vector operation, the DVM uses frequency-dependent weights defined as integer multiples of the smallest TTD component $\tau_{0}.$

### 1.1. Review: Fractional Delay Filters

Although FFT-type algorithms utilizing Thiran fractional delays are presently not available, one could observe other methods based on fractional delay filters. The authors in [12] proposed different methodologies and parameters for designing allpass fractional delay filters. The authors claimed that the employment of Lagrange FIR and Thiran fractional delay filters are the best choices for achieving desirable phase delay responses. The paper [13] evaluated designs for FIR fractional filters, including sinc, frequency sampling, polynomial interpolation, and least integral-square error. In designing fractional delay filters, the paper [14] addressed the utilization of Taylor series expansions of $e^{-j\omega \tau }$ when τ represents a combination of integer delay and a fractional delay within the interval of [−0.5, 0.5]. This approach was utilized to design fractional delay filters and gauge the efficacy of their structure in relation to the Farrow structure, primarily in terms of the storage of filter coefficients in [14]. The realization of allpass fractional delay filters was also addressed through the truncation of the power series expansion based on the denominator of the transfer function of the variable *z* at $z^{-1}=1$ [15]. The paper [16] addressed the reconstruction techniques for nonuniformly sampled band-limited signals using digital fractional delay filters using a Vandermonde structured matrix followed by a diagonal scaling. One can also find the factorization of the transfer function of the infinite impulse response (IIR) Simpson integrator for designing fractional delay filters in [17]. On the other hand, authors in [18] presented the introduction of the Hilbert transform operator, which was developed using a half-sample delay operator created through the B-spline transform interpolation and decimation. However, there is a lack of computationally efficient algorithms in the literature, despite the fact that the methods for designing Thiran fractional delay filters have been established.

### 1.2. Wideband Beamformer Structure

**Figure 1:**
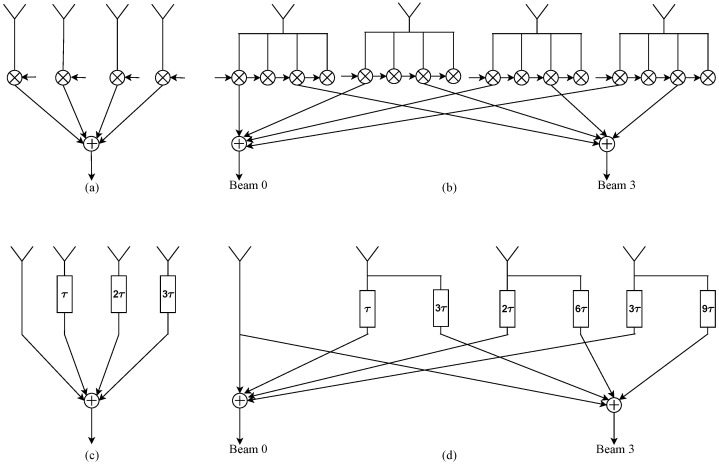
(**a**) Narrowband phased-array single-beam beamforming structure; (**b**) narrowband DFT-based multi-beam phased-array beamforming structure corresponding to a matrix–vector operation using the DFT matrix, in turn achieved via FFT based on the self-contained factorization of DFT matrix; (**c**) wideband single-beam TTD beamformer structure; (**d**) unsimplified wideband *N*-beam multi-beam TTD beamforming structure corresponding to a matrix–vector operation using the DVM. The proposed work shows how the above-mentioned architecture corresponds to a sparse factorization of DVM followed by a DVM algorithm that is realized as butterfly-like signal flow graphs (SFGs), where twiddle factors are realized in the discrete-time domain by the proposed twiddle filters based on a Thiran filter building block.

Figure 1 shows a single narrow-beam phased-array beamforming structure, a DFT-based multi-beam phased-array structure, a single TTD wideband beamformer structure, and an *N*-beam TTD wideband multi-beam structure, respectively. We first define the DVM by

$${\left[A_{N}\right]}_{kl}:=A_{N}={\left[\alpha^{kl}\right]}_{k=1,l=0}^{N,N-1},$$

where $\alpha =e^{-j\omega \tau_{0}}$. The term α accounts for the phase rotation associated with the smallest fractional delay $\tau_{0}$ (necessary for realizing the DVM N-beam system) at frequency *f*, where the temporal frequency ω=2πf. Unlike the DFT-based *N*-beam phased array, which can be realized using self-contained factorization of the DFT followed by the FFT algorithm, the corresponding *N*-beam TTD structure has no direct equivalent of the FFT. We address this problem using sparse factorization followed by a DVM algorithm while replacing complex coefficients with frequency-dependent weights realized through linear filters.

The analog realization of low-complexity and/or radix-2 DVM algorithms was previously discussed in references [7,8,9,10,11]. The structure of the DVM offers a framework for analyzing TTD-based wideband multi-beam beamforming [1]. The DVM overcomes the limitations associated with employing the DFT matrix. For example, wideband signals applied to DFT-based multi-beams lead to an undesirable “beam squint” effect. The DVM entries, referred to as $A_{N}$ in [7,8,9,10,11], serve as the foundation for a TTD-based beamformer. It is important to note that the DFT matrix equals the matrix $A_{N}$ at a single temporal frequency because the DVM covers a wide range of multiple temporal frequencies. Thus, the DVM is a superclass of the DFT matrix. However, unlike the DFT matrix, the DVM matrix lacks both properties of unitary and periodicity. Therefore, unlike the case of the DFT that can be self-factored to obtain FFT algorithms [19,20,21], we are generally unable to factorize the DVM to obtain a self-contained and radix-2 factorization, leading to an FFT-like algorithm.

### 1.3. Contribution of the Paper

To develop an efficient digital DVM algorithm that incorporates fractional delays, we will utilize the bidiagonal factorization technique in [7]. By implementing an array representation of the DVM factorization by a vector, we present a DVM algorithm that efficiently reduces the complexity associated with the matrix–matrix multiplication step of the DVM algorithm described in [7]. Furthermore, we provide SFGs specifically for small-array elements, i.e., N=4,8 incorporating Thiran fractional delays. Specifically, the fractional part associated with each node in the SFGs is implemented using Thiran fractional delay filters. Additionally, the integer part is realized using FIFO buffers implemented using D-flop pipelines and/or realized as the integer delay component of a Thiran filter. This approach allows for a precise and efficient representation of the SFGs, facilitating enhanced performance and accuracy in TTD-based wideband multi-beam digital beamforming.

### 1.4. Organization of the Paper

We provide an overview of the analog DVM algorithms proposed for TTD wideband multibeam beamforms in Section 2. In Section 3, we give an overview of digital DVM beamfomers using Thiran filters. Next, in Section 4, we recall the DVM factorization in [7] and present an array implementation of the factorization using a novel DVM algorithm. The proposed algorithm is more reliable for large *N* than that for the matrix–matrix multiplication-based DVM algorithm in [7,8]. Section 4.1 shows the arithmetic complexity (quantified using the number of necessary adders and gain–delay blocks) and numerical results for the DVM algorithm, and compares the arithmetic complexity with the matrix–vector product with the previous work. Computing the DVM–vector product with fractional delays has low arithmetic complexity compared to the brute force calculation. Section 5 presents a brief overview of analog and digital DVM beamforming using APFs. Section 6 shows the simulation for the linear phase response of Thiran filters. Next, Section 7 demonstrates Thiran filters for realizing each fractional sample delay based on the proposed DVM algorithm followed by the SFGs. Section 8 discusses future work on utilizing the APF-based Thiran fractional delay units to realize the full DVM factorization. Finally, Section 9 concludes the paper.

## 2. Review: Analog DVM Beamformers

In prior work, we proposed a sparse factorization of the *N*-beam DVM leading to 60% reduction in integrated circuit complexity [7]. This low-complexity *N*-beam DVM algorithm is based on the product of complex 1-band upper and lower matrices. In this paper, we expand on the algorithmic results in [22,23,24] by utilizing complex nodes instead of real nodes. The 1-band upper and lower matrix factorization of $A_{N}$ in [7] is expressed without utilizing quasi separability, displacement equations, and the factorization of Hankel/Toeplitz matrices [25,26,27,28,29,30,31]. Moreover, the paper addressed error bounds and stability by filling the gaps in [22,23,24] to compute DVM by a vector.

The paper [10] proposed an exact, efficient (i.e., more efficient than brute force multiplication of the DVM by a vector but not radix-2), and self-recursive DVM algorithm by using a matrix factorization of the DVM and a polynomial evaluation associated with its nodes to analyze multi-beam antenna arrays. The exact calculation of the DVM vector product via the DVM algorithm in [10] can be used to reduce the complexity of RF *N*-beam analog beamforming systems. Although the algorithms proposed in [8,10] are much more efficient than brute-force calculation, their order of arithmetic complexity is not O(NlogN). We recall here that the stable (well conditioned) and O(NlogN) algorithms for Vandermonde matrices proposed in [9] can be used for the narrowband communication system. Derivations have been established for radix-2 and split-radix FFT algorithms in [19,20,21,32,33,34,35,36]. Despite the derivation of size-*N* DFT into two size-$\frac{N}{2}$ DFTs being quite straightforward, its extension to the DVM is cumbersome because useful DFT matrix properties (periodicity and unitary) are not present in the DVM.

To overcome the above challenge, we proposed an O(NlogN) DVM algorithm having sparse factors to achieve multi-directional TTD wideband beamforming to solve the longstanding beam squint problem in [11]. In [11], the “multiplication counts” in the frequency domain—albeit in the analog domain—are a combined result of analog gains (amplifiers) and time delays in the SFG when realized in a time-domain circuit.

## 3. Digital DVM Beamfomers Using Thiran Filters

**Figure 2:**
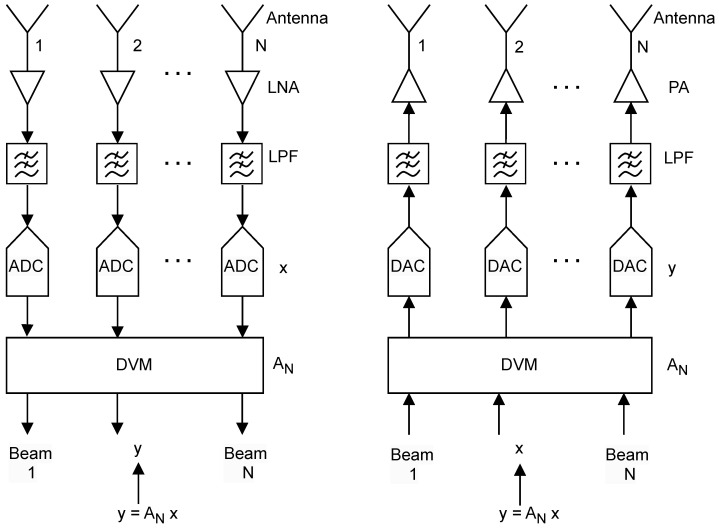
Overview architecture. RF antenna array used for optimal electromagnetic sensing of wideband plane waves based on their DOAs. (**Left**) *N*-beam TTD receiver aperture using DVM beamforming, and, (**right**) *N*-beam TTD transmit aperture using DVM beamforming.

Figure 2 shows a TTD multi-beam beamforming aperture for receive (left) and transmit (right) applications. In both cases, the beamformers are realized in the digital signal processing (DSP) unit that implements the DVM–vector operation once every sampling period. The TTD digital beamformers can be realized as the DVM–vector product s.t. $A_{N}x$, where x is the N− point vector of input signals obtained from *N* receivers (or feeding *N* transmitters after digital-to-analog conversion). In the DVM $A_{N}$, the $k^{th}$-row and $l^{th}$-column element is of the form $e^{-j\omega \tau_{0}kl}$, and hence the corresponding delays take the form $T\left(l\right)=\tau_{0}kl$, where $\tau_{0}$ is the smallest fractional delay necessary for realizing the DVM N-beam system, and ω is the temporal frequency. Typically, $\tau_{0}=T_{s}/N$. We note here that the factorization of the N-beam DVM leads to a range of delays $T_{i}=\tau_{0}P_{i}$, where *i* is the index to the $i^{th}$-node of the signal flow graph (which is, in fact, the butterfly diagram for the radix case) corresponding to the factored version of $A_{N}$ and $P_{i}\in Z^{+},$ and $P_{i}\leq {\left(N-1\right)}^{2}$ are the different time delays across the nodes of the signal flow graph (butterfly for the radix case) pertaining to the proposed fast algorithm for $y=A_{N}x$. It is important to note that at node *i*, the delay can be decomposed to the form $\tau_{0}P_{i}=n_{i}T_{s}+\tau_{i}$, where $\tau_{i}<T_{s}$ and where $n_{i}\in Z^{+}$ is an integer delay realized through FIFO register buffers located at point *i* of the signal flow graph (butterfly for the radix case). Each delay operation at node *i* consists of an integer component and a fractional component. The fractional delays $\tau_{i}\leq T_{s}$ will be realized as fast interpolation filters based on finite impulse response (FIR) and/or all-pass IIR Thiran filters. We start with the ${n_{i}}^{th}$-order Thiran fractional delay filter of the form [12]:

(1) $$H_{i}\left(z\right)=z^{-n_{i}}\frac{p_{i}\left(z\right)}{p_{i}\left(z^{-1}\right)},$$

where $p_{i}\left(z\right)=1+a_{1}z+\cdots +a_{n_{i}}z^{n_{i}-1}$, ak=(−1)kni−1k∏l=0ni−1Di−ni+lDi−ni+k+l, $D_{i}=\frac{\tau_{i}}{T_{s}}$, fractional sample delay $\tau_{i}$, temporal sample period $T_{s}$, and $k=1,2,\cdots ,n_{i}$. Here, each set of coefficients $a_{k}$ is unique to node *i*; however, we dropped the dependence on *i* for readability. We obtain an approximated transfer function for the digital all-pass Thiran fractional delay filter. Thus, with this and from the prior work on DVM [7,8,9,10,11], we propose a low-complexity wideband TTD digital DVM algorithm using Thiran fractional delays.

## 4. A Fast DVM Algorithm for Thiran Fractional Delays

We begin this section by recalling the sparse factorization of the DVM in [7]. Subsequently, an algorithm is presented based on the array implementation as opposed to the matrix–matrix implementation associated with the phase rotation $\alpha =e^{-j\omega \tau_{0}}$ followed by the smallest fractional delay $\tau_{0}$. We note that the primary objective of this study is to propose a low-complexity DVM algorithm yielding to compute DVM–vector multiplication using the Thiran fractional delays. Our work builds upon the studies [7,8,9,10,11]. It is important to note that this paper does not address related issues, such as the computation of Vandermonde structured matrices by a vector, inverse Vandermonde structured matrices (leading to inversion formulas or inversion algorithms), or the solution of the linear systems associated with Vandermonde structured matrices as the coefficient matrices as in [37,38,39,40,41,42,43,44,45,46,47].

**Proposition** **1** (Recalling from [7]). *Let the DVM, denoted by $A_{N}$, be defined via the nodes $\{\alpha ,\alpha^{2},\ldots ,\alpha^{N}\}\in C$ for integers N s.t. N≥4. Then, the DVM can be factored into the product of bidiagonal matrices s.t.*

(2) $$A_{N}=L^{\left(1\right)}L^{\left(2\right)}\cdots L^{\left(N-1\right)}U^{\left(N-1\right)}\cdots U^{\left(2\right)}U^{\left(1\right)},$$

*where*

$$\begin{array}{c} \begin{array}{cc} & L^{\left(m\right)}=\\ & \left[\begin{array}{cccccc} I_{N-m-1} & & & & & \\ & 1 & & & & \\ & 1 & \alpha^{N-m}\left(\alpha -1\right) & & & \\ & & 1 & \ddots & & \\ & & & \ddots & \ddots & \\ & & & & 1 & \alpha^{N-m}\left(\alpha^{m}-1\right) \end{array}\right], \end{array} \end{array}$$

$$U^{\left(m\right)}=\left[\begin{array}{cccccc} I_{N-m-1} & & & & & \\ & 1 & \alpha & & & \\ & & 1 & \alpha^{2} & & \\ & & & \ddots & \ddots & \\ & & & & \ddots & \alpha^{m}\\ & & & & & 1 \end{array}\right],$$

*m=1,2,⋯,N−1, and $I_{k}$ is the k×k identity matrix.*

**Remark** **1.** *We note here that the above factorization can be used to propose the ddvm algorithm (Algorithm 1) based on Thiran fractional delays when the nodes of the DVM (i.e., $\alpha^{k}$) are associated with the phase rotation $\alpha =e^{-j\omega \tau_{0}}$ followed by the smallest fractional delay $\tau_{0}$. Additionally, in Section 5, we utilize the above factorization to present the signal flow graphs for $y=A_{N}x$, where the (k,l) element of the matrix $A_{N}$ is determined by the fractional delay component associated with $e^{-j\omega \tau_{0}kl}$.*

**Algorithm 1** *ddvm*
**Input:** Integer *N* s.t. N≥4, $\alpha =e^{-j\omega \tau_{0}}\in C$ having the smallest fractional delay $\tau_{0}$, and $x\in R^{N}$ or $C^{N}$**Output:** $y=A_{N}\cdot x$**Function:** y=ddvm(x,N,α)**Steps**:**Set** s=2N, $u={\left[\begin{array}{cccc} \alpha & \alpha^{2} & \cdots & \alpha^{N} \end{array}\right]}^{T},$ and $A=\left[\begin{array}{cc} x & 0_{N\times (s-1)} \end{array}\right].$**for** l=1,2,⋯,N−1,**for** k=1,2,⋯,N, $\begin{array}{c} \mathrm{if}\;\;((k<(N-l))\;\;\mathrm{or}\;\;(k=N))\\ A(k,l+1)=A(k,l),\\ \mathrm{else}\\ A(k,l+1)=A(k,l)\\ \;+A(k+1,l)\cdot u(k-N+l+1),\\ \mathrm{end}\;\;\mathrm{if}.\\ \mathrm{end}\;\;\mathrm{for},\\ \mathrm{end}\;\;\mathrm{for}. \end{array}$**for** l=N,N+1,⋯,s−1,**for** k=1,2,⋯,N, $\begin{array}{c} \mathrm{if}\;\;(k\leq (l-N+1))\\ A(k,l+1)=A(k,l),\\ \mathrm{else}\\ A(k,l+1)=A(k-1,l)\\ \;+A(k,l)\cdot (u(k)-u(l-N+1)),\\ \mathrm{end}\;\;\mathrm{if}.\\ \mathrm{end}\;\;\mathrm{for},\\ \mathrm{end}\;\;\mathrm{for}. \end{array}$**return** A(:,s), and **Set** y=A(:,s).


### 4.1. Arithmetic Complexity of the DVM Algorithm

We show that the arithmetic complexity of the proposed DVM algorithm is less than the standard quadratic complexity. Furthermore, when the algorithm executes, it uses array implementation to avoid matrix–matrix multiplication in [7]. The number of additions and multiplications used to execute the DVM algorithm correspond to the adders and gain delay blocks, respectively.

The digital DVM using Thiran fractional delays describes a DSP system where each fractional true time delay is realized using an IIR all-pass fractional delay filter. The Thiran filter is one type of digital all-pass filter that maintains nearly constant group delay in the frequency domain −π/2≤ω≤π/2. The Thiran filters in this work will be realized using the direct-form type-II SFG so that the amount of D-flop FIFO memory is minimized.

The number of complex additions (say #a) and the number of complex multiplications (say #m), i.e., adders and gain–delay blocks, respectively, are required to compute the DVM algorithm, i.e., *ddvm*, stated next.

**Lemma** **1.** *Let N≥4 be a given integer and $x\in C^{N}$. The DVM algorithm, i.e., ddvm, can be computed with the following arithmetic complexities (respectively, adders and gain–delay blocks):*

(3) $$\begin{array}{cc} \#a(ddvm,N) & =\frac{3}{2}N\left(N-1\right),\\ \#m(ddvm,N) & ={\left(N-1\right)}^{2}. \end{array}$$

**Proof.**  To pre-calculate the vector u in Step 1 of the algorithm, one has to use $\frac{N(N+1)}{2}$ gain–delay blocks but no adders. These counts are based on pre-computation and correlated with the design time computations. Also, the aim of Step 1 is to compute the output of the algorithm effectively. In Step 2 of the algorithm, there are no adders or gain–delay counts involved in the *if* part. For the *else* part, when (N−k)≤i≤(N−1), we have one adder and gain–delay for each *k*, so in total, we have $\frac{N(N-1)}{2}$ adders and also gain–delay blocks in Step 2. In Step 3 of the algorithm, there are no adders or gain–delay involved in the *if* part. For the *else* part, when (k−N+1)<i≤N, we have two adders and one gain–delay for each *k*, so in total we have N(N−1) adders and $\frac{N(N-1)}{2}$ gain–delay blocks in Step 3. In Step 4 of the algorithm, we extract the last column of A and assign it to the vector y; hence, no cost is involved. Hence, the proposed algorithm requires $\frac{3}{2}N\left(N-1\right)$ adders (respectively, complex additions) and ${\left(N-1\right)}^{2}$ gain–delay blocks (respectively, complex multiplications). □

**Remark** **2.** *We recall here that an array implementation of a DVM algorithm was used to solve the DVM system for the phased array digital receivers in [45], and hence were able to reduce the cost of solving the DVM system from $O(N^{3})$ to $O(N^{2})$. Unlike in [45], we do not solve the systems of equations but rather compute the DVM–vector product.*

### 4.2. Comparison Results for the Arithmetic Complexities of DVM Algorithms

In this section, we compare the arithmetic complexity of the proposed DVM algorithm with the related work of DVM algorithms.

The gain–delay blocks of the proposed DVM algorithm (i.e., ${\left(N-1\right)}^{2}$) show favorable results in comparison to [7] (i.e., $\frac{N(3N-1)}{2}$). This advantage is gained based on the pre-computation of nodes followed by the array implementation of the proposed algorithm. If we consider the gain–delay blocks in the pre-computation stage of the ddvm algorithm (step 1), the number of gain–delay blocks in the proposed algorithm and the algorithm in [7] is the same, i.e., $\frac{3}{2}N^{2}+O\left(N\right)$. Furthermore, the number of adders remains consistent between the ddvm algorithm and the algorithm in [7]. The ddvm algorithm exhibits a higher count of adders and gain–delay blocks compared to the algorithms presented in [10,11]. This is because the algorithms in those papers have adders and gain–delay block counts of the order O(NlogN), whereas the ddvm algorithm maintains these counts of less than $O(N^{2})$. But, on the other hand, it is important to note that the paper [10] introduces additional criteria for the arrangement of nodes. Specifically, the nodes are evenly distributed on a circle centered at the origin with a radius *r*, where r≥1. This criterion is not imposed in this paper. Despite the fact that the algorithms presented in [11] exhibit a complexity of O(NlogN), this algorithm does not yield favorable outcomes when applied to antenna arrays with a small number of elements, specifically 4 and 8. However, the DVM algorithm in [11] shows significant efficiency, surpassing 90%, for DVM sizes of N≥256. On the other hand, the gain–delay blocks of the ddvm algorithm consistently exhibit better performance compared to those in [9] for both N=4 and N=8 configurations.

However, the objective of this paper is to utilize the DVM factorization, as outlined in Proposition 1 from [7], for the implementation of multi-beam digital wideband beamforming systems that employ Thiran fractional delays. Thus, we will utilize the proposed algorithm to realize the signal flow graphs as shown in the next section.

## 5. Thiran Fractional Delays for Twiddle Filter Realization

In this section, we will provide a brief overview and formulation of how analog and digital DVM beamforming can be realized using APFs.

### 5.1. Analog RC Lattice APFs

The linear phase shift associated with analog DVM beamforming is $e^{-j\omega \tau }$, where $\tau >\tau_{0}$. The factor $e^{-j\omega \tau }$ is associated with the factorization of the $A_{N}$ and can be efficiently approximated on chip by CMOS APFs [7,8,9,10,11,48,49].

### 5.2. Digital Fractional Delay APFs

Upon digitization, the time domain becomes discrete due to the sampling operation in the ADCs. Hence, the linear phase fractional sample delay blocks corresponding to the (k,l) element of $A_{N}$, i.e., $e^{-j\omega \tau_{0}kl}$ where $\tau_{0}$ is the smallest fractional delay necessary for realizing the DVM N-beam DSP system. The matrix factorization of $A_{N}$ for digital DVM can be realized using discrete-domain Thiran fractional delay filters while approximating the fractional time delay, i.e., $\tau_{i}<T_{s}$ where $T_{s}=\tau_{0}N$.

### 5.3. Thiran IIR Filter Blocks

Unlike the analog DVM case, which uses the fact that $e^{-j\omega \tau_{i}}\approx {\left(\frac{1-j\omega \tau_{i}/2M}{1+j\omega \tau_{i}/2M}\right)}^{M},\;M\in Z^{+}$ and ω is the frequency variable in general, the digital DVM requires Thiran fractional delay approximations given by the digital infinite impulse response (IIR) filter function $H_{i}\left(z\right)$, having a filter order $n_{i}\in Z^{+}$. The purely fractional delay component ψ(z) is related to the Thiran filter via $H_{i}\left(z\right)=z^{-n_{i}}{\left(\psi \left(z\right)\right)}^{n_{i}}$, and ψ(z) is the fractional delay associated with $\tau_{0}.$

### 5.4. Higher-Order Thiran APFs

We note that the fractional delay ${\left(\psi \left(z\right)\right)}^{p}$ can be realized either by cascading *p* units of first-order (or low-order) digital ψ(z) all-pass filters or by designing a single APF with the desired level of fractional delay so that it fits the model of $H_{i}\left(z\right).$ The former approach leads to *p*-times replicated computational hardware structures of a Thiran filter when realized as a VLSI implementation. We adopt the latter approach, where a unique Thiran filter of order $n_{i}$ is realized as a hardware core for the implementation of $\psi {\left(z\right)}^{p}$.

## 6. Simulation of Thiran Fractional Delays

**Figure 3:**
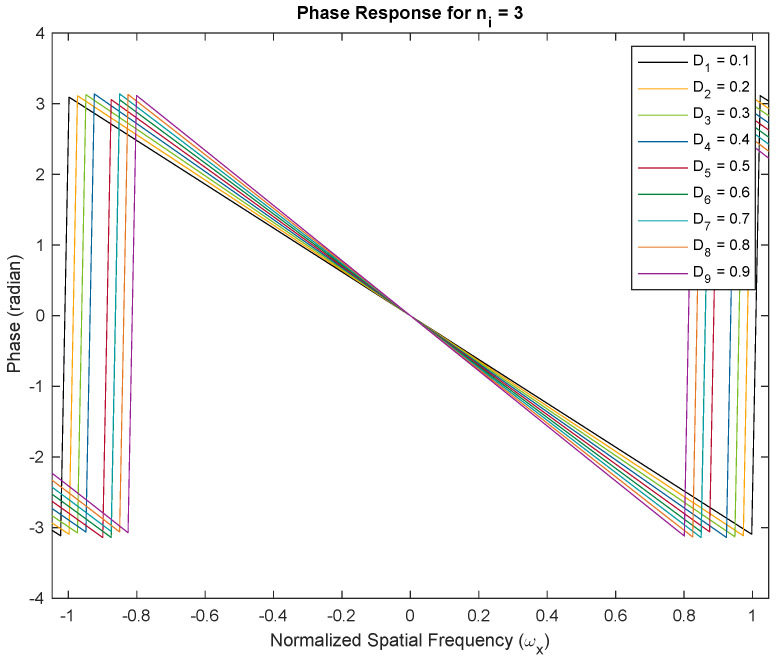
Phase responses for order $n_{i}=3$ and delay variations $D_{i}=0.1$–0.9, where $D_{i}=\tau_{i}/T_{s}.$ With temporal over-sampling of 3×, the usable range of the Thiran filter response is contained within the normalized −0.33 to 0.33 frequency band. The linear phase response establishes the role of Thiran filters in realizing fractional sample delays.

**Figure 4:**
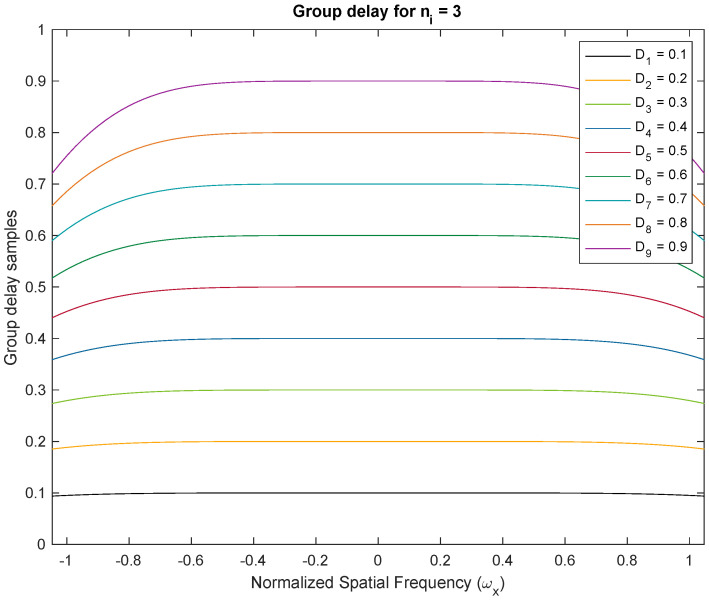
Group delay profiles for order $n_{i}$ = 3 and delay variations $D_{i}=0.1$–0.9, where $D_{i}=\tau_{i}/T_{s}$. Constant group delay can be observed over 50% of the Nyquist interval. With temporal over-sampling of 3×, the usable range of the Thiran filter response is contained within the normalized −0.33 to 0.33 frequency band. The constant group delay establishes the role of Thiran filters for realizing fractional sample delays.

**Figure 5:**
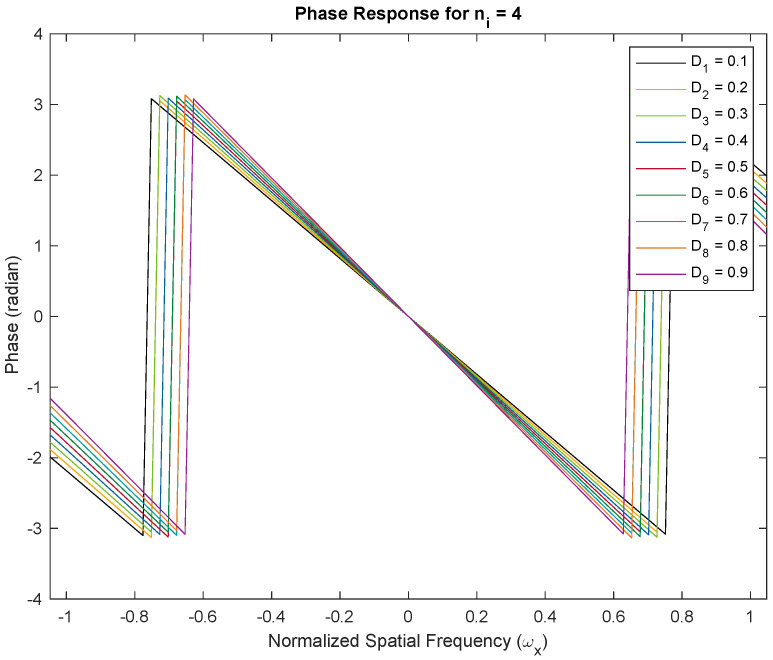
Phase responses for order $n_{i}=4$ and delay variations $D_{i}=0.1$–0.9, where $D_{i}=\tau_{i}/T_{s}$. With temporal over-sampling of 3×, the usable range of the Thiran filter response is contained within the normalized −0.33 to 0.33 frequency band. The linear phase response establishes the role of Thiran filters in realizing fractional sample delays.

**Figure 6:**
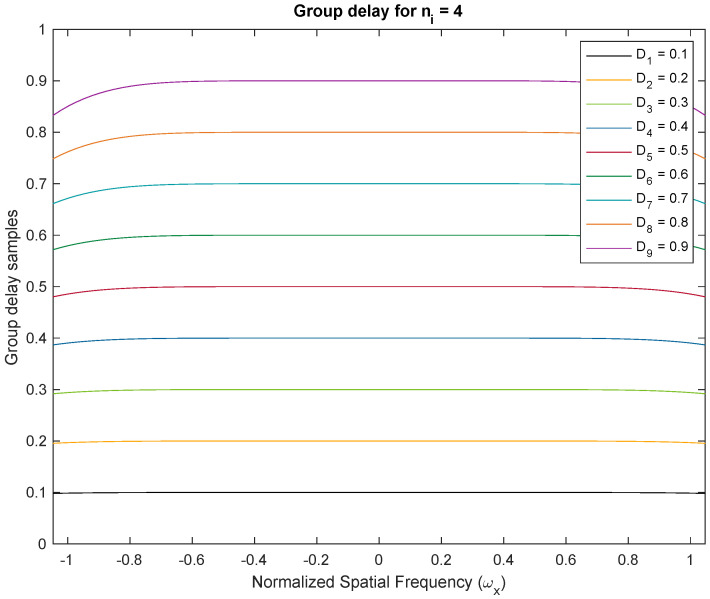
Group delay profiles for order $n_{i}=4$ and delay variations $D_{i}=0.1$–0.9. Constant group delay can be observed over 50% of the Nyquist interval. With temporal over-sampling of 3×, the usable range of the Thiran filter response is contained within the normalized −0.33 to 0.33 frequency band. The constant group delay establishes the role of Thiran filters in realizing fractional sample delays.

We demonstrate the approximately linear phase response of Thiran filters. We show two examples with fractional delays of $D_{i}=0.1$–0.9 fractions of the temporal sampling period. We used Thiran IIR APFs of order $n_{i}=3$ and $n_{i}=4,$ respectively, with corresponding integer delay components of three and four clock periods, and the desired fractional delay components. Both filters are usable as approximately linear-phase fractional sample delay blocks better than the first 50% of the Nyquist period corresponding to a temporal oversampling factor of about two. All plots of the phase and group delay vs. frequency are in the domain −π/3≤ω≤π/3. In Figure 3, we show the phase response of the Thiran all-pass filter with $n_{i}=3$ and $D_{i}=0.1$–0.9. The negative linear phase behavior of the filter within the first half of the Nyquist period is clearly apparent from the simulation. The approximately linear phase response results in approximately constant group delays as shown in Figure 4. The experiments are repeated for $n_{i}=4$ in Figure 5 for phase responses and Figure 6 for group delay, respectively.

### Phase Responses and Group Delay Profiles of Thiran Filters

Figure 3 and Figure 5 illustrate phase responses for order $n_{i}=3$ and order $n_{i}=4$ for delay variations $D_{i}=0.1$–0.9, where $D_{i}=\tau_{i}/T_{s}$ respectively. Figure 4 and Figure 6 illustrate the group delay profiles for order $n_{i}$ = 3 and order $n_{i}=4$ for delay variations $D_{i}=0.1$–0.9, where $D_{i}=\tau_{i}/T_{s}$ respectively.

**Figure 7:**
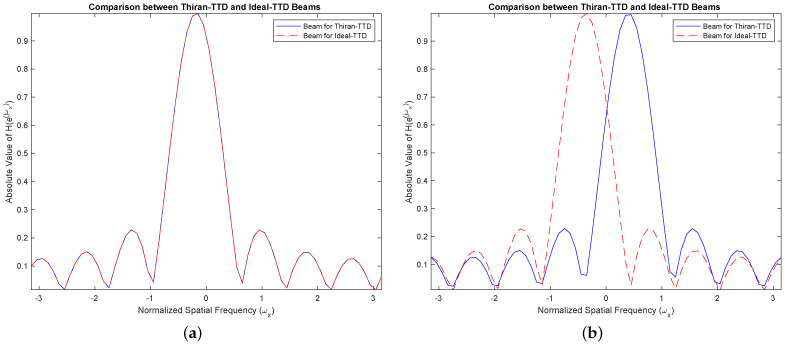
(**a**) shows a TTD beam for 3× temporal over-sampling (ω=0.33π) which is the worst-case performance under the recommended scheme. The Thiran filter yields a beam that is identical to the perfect delay case. Furthermore, we show in (**b**) what would happen when the frequency is out of the recommended range, i.e., when ω=0.8π, where there is a significant deviation of the beam axis. We only show this for information. In practice, by keeping ω<0.33π by selecting 3× temporal over-sampling, we can avoid this issue.

The TTD beam shapes of the multi-beam beamformer remain unchanged regardless of the method used to achieve the TTDs. The main requirement is to have a constant group delay that matches the desired TTD value and a response with a unit magnitude. These conditions are satisfied within the usable frequency range. The group delay of the Thiran filters enables the desired TTD value to be achieved within the operating band, assuming temporal over-sampling up to 3×. If the group delay of the TTD at a specific frequency matches the desired TTD, the aperture will produce the same beam. When there is an insufficient amount of temporal oversampling, the Thiran filters may not perform at their best, making a strong case for deviation. In practice, we can disregard this due to the use of significant over-sampling. Our paper concludes that the Thiran filter-based approach is exclusively suitable for temporal over-sampling factors greater than approximately 3× and summarizes this using Figure 7.

## 7. SFGs for the Fast Digital DVM Algorithm Using Thiran Fractional Delays

In this section, we will utilize SFGs to elaborate on Thiran fractional delays for twiddle filter realization. Although a direct-form realization of the digital DVM beamformer [50] can be realized using Thiran filters for realizing each fractional sample delay, we proposed to reduce the computational complexity of the multi-beam beamformer by exploiting sparse factorization of the DVM in [7] followed by the fast DVM algorithm, i.e., ddvm. However, the SFGs in [7] are continuous-time and realized as analog microwave circuits.

In this paper, we are operating in the discrete domain, and the resulting SFGs are discrete-time and realizable using DSP systems; to wit, the sparse factorization of the DVM leads to discrete-time SFGs having twiddle filters consisting of digital fractional delay lines that can be approximated using Thiran filter building blocks.

### 7.1. An N=4 Point DVM with Thiran Twiddle-Filters

**Figure 8:**
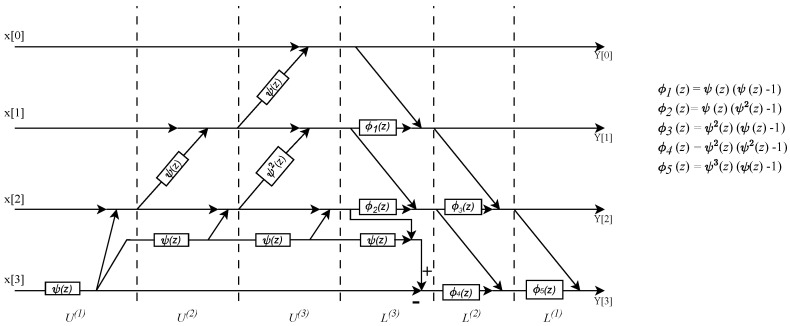
SFG for the N=4–beam wideband beamformer using the DVM factorization followed by the *ddvm* algorithm, having the fractional delays ψ(z) associated with $\tau_{0}$.

**Figure 9:**
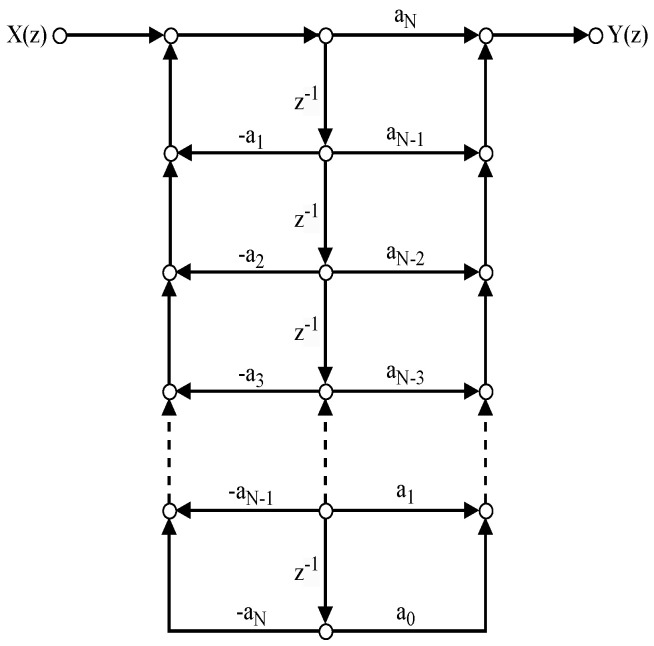
The direct form II SFG of a Thiran filter designed to achieve a fractional true time delay in the first Nyquist zone.

**Figure 10:**
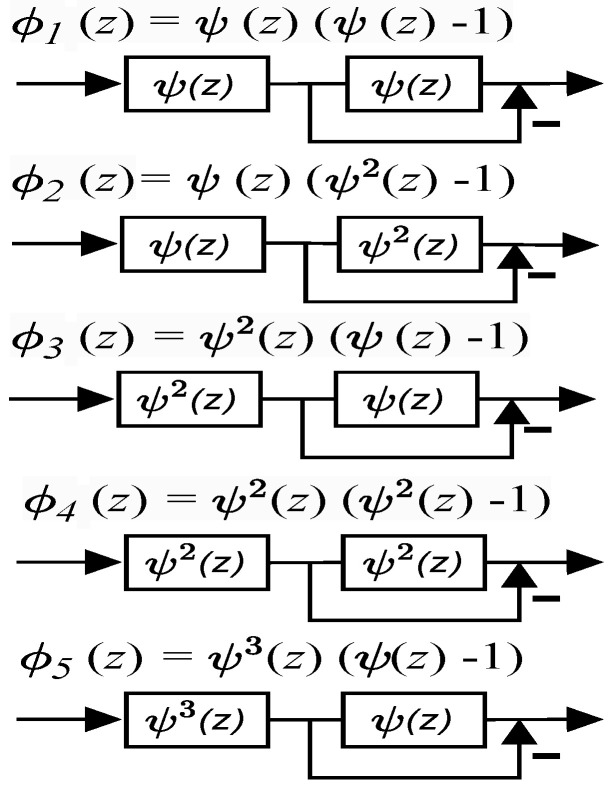
$\phi_{k}\left(z\right)$ function architectures for the order N=4 filter. It showcases the reuse of the ψ(z) Thiran block as a fundamental building block for creating these specialized filters.

Figure 8 shows the N=4 point DVM factorization, where fractional true time delays of the form $\psi^{p}\left(z\right)$ are realized as a single Thiran filter block, having delay pτ where τ=1/N. Figure 9 shows the direct form II SFG of a typical Thiran all-pass filter that approximates a fractional true time delay within the first 60% of the Nyquist period. A temporal over-sampling factor of two easily allows the signals of interest to be located within the usable band of the Thiran filters of the transfer function order $n_{i}=3$ and $n_{i}=4$, respectively. The twiddle filter-based DVM factorizations also require several special filters denoted as $\phi_{k}\left(z\right),k=1,2,...,5$, where each special filter is realized using ψ(z) Thiran filters as a building block. Therefore, once the Thiran block ψ(z) is available, it can be re-used to create the special twiddle filters $\phi_{k}\left(z\right)$ as shown in Figure 10.

### 7.2. An N=8 Point DVM with Thiran Twiddle-Filters

**Figure 11:**
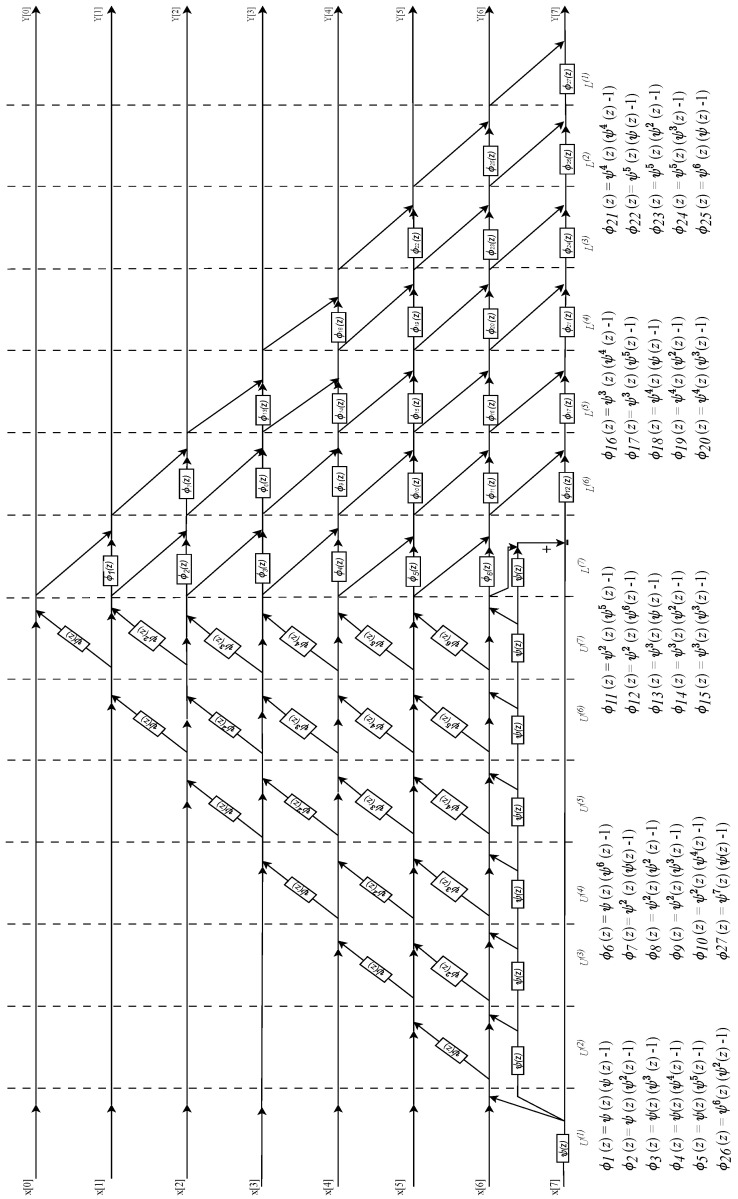
SFG for the N=8–beam wideband beamformer using the DVM factorization followed by the *ddvm* algorithm having the fractional delays ψ(z) associated with $\tau_{0}$.

**Figure 12:**
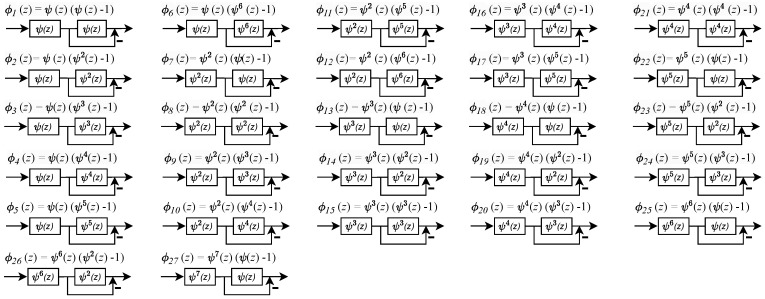
$\phi_{k}\left(z\right)$ function architectures for the order N=8 filter. The twiddle filters are realized using the ψ(z) Thiran filter hardware core as a building block.

Figure 11 shows the N=8 point DVM factorization, where fractional true time delays are of the form $\psi^{p}\left(z\right)$ as was the case for N=4. For N=8, the twiddle filters require special filters that also use ψ(z) Thiran filters as the primary building block. The twiddle filters $\phi_{k}\left(z\right)$ are realized using ψ(z) Thiran fractional delay filters as the building blocks. Once a reconfigurable Thiran filter is realized in a hardware design, it can be re-used at scale for realizing the SFGs consisting of the twiddle filters $\phi_{k}\left(z\right)$ using cascade and parallel-form filter synthesis as shown in the corresponding design equations for $\phi_{k}\left(z\right).$ The special filters $\phi_{k}\left(z\right)$ are shown as SFGs in Figure 12, where $\psi^{p}\left(z\right)$ are direct form II Thiran filter blocks that implement true-time fractional delays having value pτ, p=1,2,3...,6.

### 7.3. Preliminary FPGA Digital Hardware Architectures

The full realization of the DVM architecture on a digital integrated circuit (IC) is a complex task and is beyond the scope of this paper. Nevertheless, we provide preliminary results toward the eventual realization of the DVM beamformers on digital IC using application-specific integrated circuit (ASIC) technology.

#### 7.3.1. FPGA Realization of Thiran Filter Blocks

**Figure 13:**
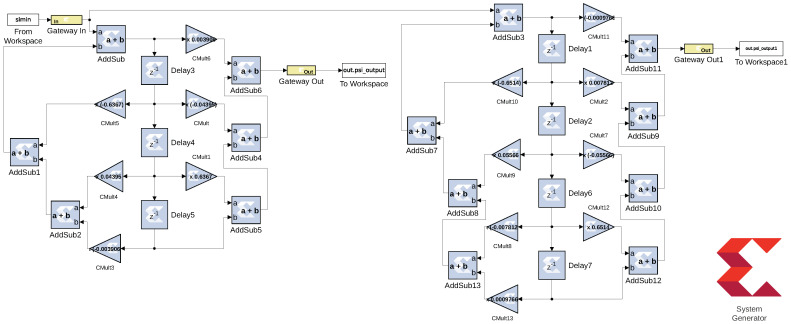
(**Left**) The direct form II computer architecture for realizing the Thiran filter block realizing ψ(z) for the order $n_{i}$ = 3. (**Right**) The direct form II computer architecture for realizing the Thiran filter block realizing ψ(z) for order $n_{i}$ = 4. The digital FPGA design uses the Xilinx System Generator FPGA blockset, which is a plug-in to Matlab/Simulink that provides a software interface to the Xilinx Vivado toolset.

We prototyped a single software reconfigurable and delay-tunable digital Thiran filter using field programmable gate array (FPGA) technology. Using Xilinx FPGA design tools, including System Generator and PlanAhead, we realized a Thiran filter as a direct form II hardware architecture. The design, shown in Figure 13, assumes that two’s complement the fixed-point computer arithmetic with a system bus size of 14 bits, and quantization of type rounding. The design was simulated for unit impulse response and compared with an ideal unit impulse response obtained from Matlab; the results match the errors associated with a 14-bit finite precision computer arithmetic core.

#### 7.3.2. Synthesis and Mapping to FPGA Fabric

The RF-SoC realization of a Thiran filter for $n_{i}=3,4$ order APFs are given in Table 1. The designs were ported from Matlab System Generator to Xilinx Vivado for synthesis and post place-and-route timing analysis. The design for order $n_{i}=3$ filter consumes 418 configurable logic block look-up tables (CLB LUTs), 158 CLB registers, and 51 CARRY8 on the Xilinx FPGA reconfigurable logic fabric. The design for the order $n_{i}=4$ Thiran consumes 487 CLB LUTs, 182 CLB registers, and 70 CARRY8 on FPGA logic fabric. The target FPGA device was an RF System-On-Chip (SoC) ZCU-111.

The hardware consumption data in Table 1 indicate the scalability of the Thiran filter building blocks for large apertures having many TTD units in the SFG.

#### 7.3.3. Post Place Route Timing

Designs were pipelined to obtain a critical path delay (CPD) of $T_{CPD}=n_{i}T_{A/S}+T_{M}$, where $n_{i}\in Z^{+}$ is the order, $T_{A/S}$ is the latency for an adder/subtractor, having 14 bits of precision, and $T_{M}$ is the latency for a multiplier, having a coefficient precision of 14 bits and requantized output precision of 14 bits, selected to match the precision of the analog-to-digital converters (ADCs) on the RF-SoC. The maximum clock frequency is $F_{CLK}=1/T_{CPD}.$ Post place and route timing analysis on Vivado indicates maximum clock speeds of 220 MHz and 213 MHz for Thiran filter orders of $n_{i}=3$ and $n_{i}=4$, respectively.

## 8. Future Work

We will utilize the APF-based Thiran fractional delay units to realize the full DVM factorizations provided in the paper. The twiddle filters will be realized using the Thiran building blocks. Thereafter, the factorized DVMs will be realized following the respective SFGs. The DVM algorithms will be pipelined using clocked D-flop stages inserted within the feed-forward parts of the signal flow. The building block Thiran filters are IIR filters that cannot be pipelined further without specialized computer architecture techniques; for example, we will explore the scattered/structured look-ahead pipelining [51] for digital processors, which are based on complex pole-zero cancellations. The computer architectures arrived at by mapping the SFGs to pipelined parallel processors with Thiran twiddle filters will be implemented on an AMD-Xilinx RF-SoC platform, such as ZCU-1285/1275/111, and operated with RF front ends realized off chip to evaluate the multi-beam beamforming performance in a hardware-in-the-loop emulation [52,53,54].

## 9. Conclusions

The ability to directionally enhance a propagating wideband RF plane-wave signal is a crucial requirement for RF sensing applications, as well as RF imaging, RF communications, and RF-based location systems. The application domains of RF sensing include radar, radio astronomy, and joint-communication and sensing applications that are emerging for next-generation wireless systems (e.g., 6G networks). A multi-beam TTD beamformer allows optimal sensing along a set of pre-determined DOAs for wideband waveforms. We proposed an algorithm for the efficient computation of wideband true time delay digital DVM using Thiran fractional delays. By adopting an array implementation of the DVM algorithm followed by the bidiagonal and diagonal matrices, the complexity of the digital DVM is significantly reduced. By applying this algorithm, the digital DVM becomes more efficient and optimized. Consequently, the algorithm yields a reduction in circuit complexity for multi-beam digital wideband beamforming systems that implement Thiran fractional delays. We implemented the DVM algorithm to effectively demonstrate the phase and group delay responses of Thiran filters that possess fractional sampling period delays of both the third and fourth orders. These filters can be integrated into the Xilinx FPGA RF-SoC technologies. To validate the feasibility of implementing the DVM algorithm, we utilized signal flow graphs as the basis for realizing digital application-specific integrated circuits (ASICs).

## Figures and Tables

**Table 1:** AMD-Xilinx ZCU-111 RF-SoC realizations of Thiran filters for orders $n_{i}=3$ and $n_{i}=4$, respectively.

Order	Site Type	Used	Available	Util %
3	CLB LUTs	418	425,280	0.10
	LUT as Logic	410	425,280	0.10
	LUT as Memory	8	213,600	<0.01
	LUT as Distributed RAM	0		
	LUT as Shift Register	8		
	CLB Registers	158	850,560	0.02
	Registers as Flip Flop	158	850,560	0.02
	Registers as Latch	0	850,560	0.00
	CARRY8	51	53,160	0.10
4	CLB LUTs	487	425,280	0.11
	LUT as Logic	471	425,280	0.11
	LUT as Memory	16	213,600	<0.01
	LUT as Distributed RAM	0		
	LUT as Shift Register	16		
	CLB Registers	182	850,560	0.02
	Registers as Flip Flop	182	850,560	0.02
	Registers as Latch	0	850,560	0.00
	CARRY8	70	53,160	0.13

## Data Availability

Data are contained within the article.

## Nomenclature

$A_{N}$	N×N DVM
ADC	Analog to digital converter
APF	All-pass filter
ASIC	Application-specific integrated circuit
AWGN	Additive white Gaussian noise
CMOS	Complementary metal-oxide semiconductor
Complex Laplace variable	s∈C
Complex *z*-variable	z∈C
Complex polynomial of order $n_{i}$	$p_{i}\left(z\right)$
CLB	Configurable logic block
Critical path delay	$T_{CPD}$
DAC	Digital to analog converter
Delay (integer and fractional parts)	τ
DFT	Discrete Fourier transform
Digital integer delays at node *i*	$P_{i}$
Digital fractional delay at node *i*	$D_{i}$
DSP	Digital signal processing
DOA	Directions of Arrival
Discrete-time APF	ψ(z)
DVM	Delay Vandemonde matrix
FFT	Fast Fourier transform
FPGA	Field programmable gate array
FIFO	first in first out
FIR	Finite impulse response
IIR	Infinite impulse response
Input vector or signals to DVM	**x**
Latency of addition/subtraction core	$T_{A}/D$
Latency of multiplier core	$T_{M}$
LNA	Low noise amplifier
LPF	Low pass filter
LUT	Look-up table
Nodes of DVM	$\alpha^{k}=e^{-j\omega \tau_{0}k}$
Number of APFs in cascade	*M*
Order of *i*-th APF	$n_{i}$
Order of DVM	N×N
Output vectors or beamformed signals	**y**
Phase function at (p,q)	$\alpha_{p,q}$
Phase rotation	$\alpha =e^{-j\omega \tau_{0}}$
RF	Radio frequency
RAM	Random access memory
Sampling period	$T_{s}$
SFG	Signal flow graph
SNR	Signal-to-noise ratio
Smallest fractional delay	$\tau_{0}$
SoC	System on chip
Temporal frequency	ω
The *i*-th node in the SFG	*i*
Thiran APF at node *i*	$H_{i}\left(z\right)$
Time delay at node *i*	$\tau_{i}$
TTD	True time delay
Twiddle Filter at node *i*	$\phi_{i}\left(z\right)$
VLSI	Very large scale integration
